# A bioinspired analogous nerve towards artificial intelligence

**DOI:** 10.1038/s41467-019-14214-x

**Published:** 2020-01-14

**Authors:** Xinqin Liao, Weitao Song, Xiangyu Zhang, Chaoqun Yan, Tianliang Li, Hongliang Ren, Cunzhi Liu, Yongtian Wang, Yuanjin Zheng

**Affiliations:** 1 School of Electrical and Electronic Engineering, Nanyang Technological University, 50 Nanyang Avenue, Singapore, 639798 Singapore; 2 Department of Acupuncture and Moxibustion, Dongzhimen Hospital, Beijing University of Chinese Medicine, Hai Yun Cang on the 5th Zip, Beijing, 100700 China; 3 Department of Biomedical Engineering, National University of Singapore, 21 Lower Kent Ridge Road, Singapore, 119077 Singapore; 4 Beijing Engineering Research Centre of Mixed Reality and Advanced Display, School of Optics and Photonics, Beijing Institute of Technology, 5 Zhongguancun South Street, Beijing, 100081 China; 5 AICFVE of Beijing Film Academy, 4 Xitucheng Road, Beijing, 100088 China

**Keywords:** Electrical and electronic engineering, Sensors and biosensors

## Abstract

A bionic artificial device commonly integrates various distributed functional units to mimic the functions of biological sensory neural system, bringing intricate interconnections, complicated structure, and interference in signal transmission. Here we show an all-in-one bionic artificial nerve based on a separate electrical double-layers structure that integrates the functions of perception, recognition, and transmission. The bionic artificial nerve features flexibility, rapid response (<21 ms), high robustness, excellent durability (>10,000 tests), personalized cutability, and no energy consumption when no mechanical stimulation is being applied. The response signals are highly regionally differentiated for the mechanical stimulations, which enables the bionic artificial nerve to mimic the spatiotemporally dynamic logic of a biological neural network. Multifunctional touch interactions demonstrate the enormous potential of the bionic artificial nerve for human-machine hybrid perceptual enhancement. By incorporating the spatiotemporal resolution function and algorithmic analysis, we hope that bionic artificial nerves will promote further development of sophisticated neuroprosthetics and intelligent robotics.

## Abstract (web summary)

Designing artificial sensory neural system for neuroprosthetics and smart robotics applications remains a challenge. Here, the authors demonstrate an all-in-one bionic sensory transmission nerve capable integrating the functions of perception, recognition and transmission of mechano-sensitive signal.

## Introduction

Sensory neurons within the skin provide an easy and intuitive interface to convert the external stimulation into a physiological signal for touch recognition and brain learning^1^. Crucially, the development of artificial sensory neural systems helps to restore touch perception of disabled persons^2–5^, construct a hybrid bioelectronic reflex arc to actuate muscles^6^, and build interactive feedback of robotic manipulation^7,8^. Notable advancement is obtained in the bionic sensor^9–14^, signal cable^15–17^, and synaptic transistor^18–22^, which are the essential and main functional elements of the artificial sensory neural system. Alternatively, a highly sensitive tactile sensor based on a giant magneto-impedance material with a rational structural design was functionalized in a low-pressure regime^23^. To mimic the signal transport function of axons, an ionic cable was therefore proposed to transmit signal at high speed over a long distance^24^. For building a neuromorphic system, a laterally coupled indium-zinc-oxide-based artificial synaptic transistor was successfully fabricated to demonstrate spatiotemporal signal processing^25^. Although the great achievements have been made, most reported bionic sensory devices were integrated with discrete elements, which will require intricate interconnections and complicated structure, and may induce interference in signal transmission. Furthermore, the bionic sensory device should be low or no energy consumption to work as a biological synapse when there is no touch^26^. Pursuing the softness that endows mechanical compliance of bionic sensory device like a natural nerve still faces challenges.

Here we propose an all-in-one bionic sensory transmission nerve to achieve the above goals. To demonstrate this concept, we fabricated an artificial perception and transmission nerve (APT nerve). The APT nerve was designed by adopting a separate electrical double-layers structure. Our design principle was based on electric contact theory^27^, which was the key to detect external mechanical stimulation such as finger or object touch. Based on the proposed structure, the APT nerve featured no energy consumption except that mechanical stimulation occurs. The biological nerve fibers feature a long strip morphology and are flexible in biological tissue^28^. Inspired by the biological morphology, the architecture of the APT nerve was initially designed as a long strip based on the separate electrical double-layers structure. Due to the universality of the design principle, in addition to the linear strip shape, the architecture of the APT nerve could be designed into a wide variety of prototype devices as needed, including L-shaped, S-shaped and square devices. This architecture enabled the APT nerve to further recognize the location of external mechanical stimulation without requiring the integration of multiple sensing units. Moreover, the extended strip-shaped architecture realized the APT nerve integrating the functions of mechano-perception and signal transmission. In order to achieve the characteristic of flexibility, a fiber-based paper was selected as the substrate to fabricate the APT nerve. Previous studies showed that a conductive film, which consisted of plentiful graphite slices, could be formed by pencil drawing on the paper-based substrate^29^. Overlaps or cracks were generated among the graphite slices by bending the paper-based substrate inward or outward so that the resistance of the conductive film would decrease or increase correspondingly. In our design, the APT nerve consisted of two graphite-based conductive films, which were face-to-face taped together and served as the active layers. Bending the APT nerve would make the upper and bottom active layers bent oppositely so that the changes in the resistance of the two active layers were reversed. As a result, the overall resistance of the APT nerve would be relatively stable and thus, the APT nerve could operate in bending state. In addition, the proposed APT nerve based on the bionic architecture also featured high stability, rapid response, and even cuttability. As a proof-of-concept, we demonstrated that the APT nerve was capable of not only converting mechanical stimulation into an electrical signal but also recognizing the location of the mechanical stimulation. After logical analysis, the mechanosensitive signal could be transmitted and used for subsequent interactive control, including playing music, controlling positioning in the two-dimensional plane, and free handling rotation, which confirmed the huge potentials of the APT nerve for smart prosthetics and socially intelligent robotics. Furthermore, we explored the spatiotemporal resolution function of the APT nerve, which could be incorporated with the algorithmic analysis of artificial intelligence that would genuinely promote neuroprosthetics and neurorobotics into sophistication. We believe that the APT nerve can also be fabricated by using different functional materials that will allow the APT nerve to become stretchable, transparent, and multifunctional as needed in the future.

## Results

### Biological inspiration and design of the APT nerve

**Fig. 1:**
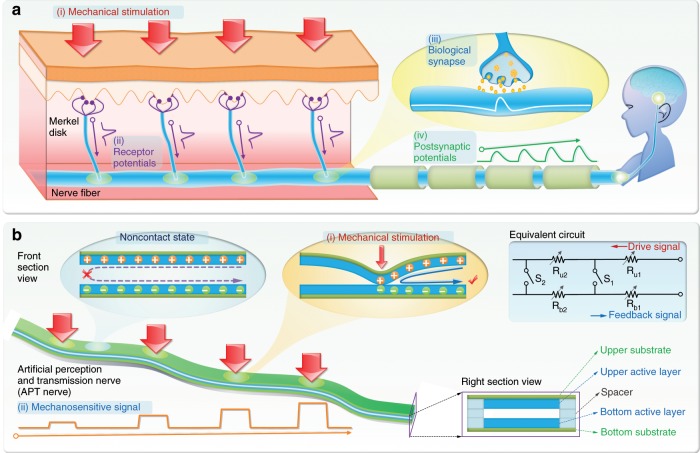
Biological sensory neurons and an artificial perception and transmission nerve. **a** Schematic of the function of biological sensory neurons. Mechanical stimulations are converted into receptor potentials by mechanoreceptors. The receptor potentials induce postsynaptic potentials by synapses. Postsynaptic potentials are transmitted to the cortex for information processing through the interneurons. **b** Architecture and working mechanism of an artificial perception and transmission nerve (APT nerve). Mechanical stimulation applied on the APT nerve is directly converted into mechanosensitive signals for information processing. Top left: Working mechanism of the APT nerve. Top right: Equivalent circuit. Bottom right: Structure of the APT nerve in right section view.

Biologically, the external mechanical stimulation is converted into receptor potentials by mechanoreceptors^30^, such as Merkel disk (MD) that detects static force at slow adaptation rate (Fig. 1a). The receptor potentials are encoded by synapses, which are formed between the multiple afferent neurons and the interneuron in the spinal cord, and then awaken the interneurons^31^. Subsequently, the encoded postsynaptic potentials are transmitted to the cortex in turn through the interneurons to recognize the location of the external mechanical stimulation^32^. Thus, the proposed APT nerve should have the four essential functions, including detecting the mechanical stimulation, consuming low or no energy unless mechanical stimulation happens, transmitting mechanosensitive signal, and recognizing the location of mechanical stimulation.

To demonstrate this concept, the proposed APT nerve was designed to emulate the functions of sensory neurons (Fig. 1b). We used a conductive graphite film, which was prepared by a carbon pencil, and a piece of graph paper to serve as the active layer and substrate, respectively, and to construct the prototype of the APT nerve (Supplementary Fig. 1). Two parts that paper-based substrate combined with the conductive graphite film were face-to-face put together with a thin spacer, which ensured that the upper and bottom active layers were separated when no external mechanical stimulation was applied on the APT nerve (Seeing the inset image at the bottom right corner of Fig. 1b). Note that the conductive graphite film was drawn on the paper-based substrate, which was compatible with flexibility and had the potential for optionally preparing different shapes of the APT nerve. In our design, the upper and bottom active layers were in the noncontact state, so that no current would flow through the APT nerve, which made sure the APT nerve did not consume energy when no mechanical stimulation was applied on it.

Among various types of sensing mechanisms, the electric contact sensing mechanism was adopted for the APT nerve. When a mechanical stimulation, such as a finger touch, was applied on the APT nerve, the upper and bottom active layers contacted together at the location of the mechanical stimulation. Subsequently, the electric contact caused the mechanosensitive signal to flow from the upper active layer to the bottom active layer. The simplified equivalent circuit was shown at the top right corner of Fig. 1b. Here, the APT nerve was equivalent to a device consisting of numerous resistor units, and the upper and bottom resistors were disconnected by switches. When there was an external mechanical stimulation, the corresponding switch would be closed so that the APT nerve had a corresponding response resistance. The response resistance (*R*_*total*_) could be expressed as the following equation:

1 $$R_{total} = \mathop {\sum }\limits_{i = 1}^n (R_{u1} + R_{b1} + \ldots + R_{ui} + R_{bi})$$

where *R*_*ui*_ and *R*_*bi*_ were the resistance of the virtual resistor units of the upper and bottom active layers, respectively. Thus, the magnitude of the response resistance was determined by the location of the mechanical stimulation. As the location moved away from the electrodes, the response resistance would increase. By analyzing the magnitude of the response resistance, the APT nerve could recognize the location of the mechanical stimulation. The proposed APT nerve was limited to transmitting and recognizing one location at a time that was analogous to the function of the interneuron in the spinal cord.

### Performance and characteristic of the APT nerve

**Fig. 2:**
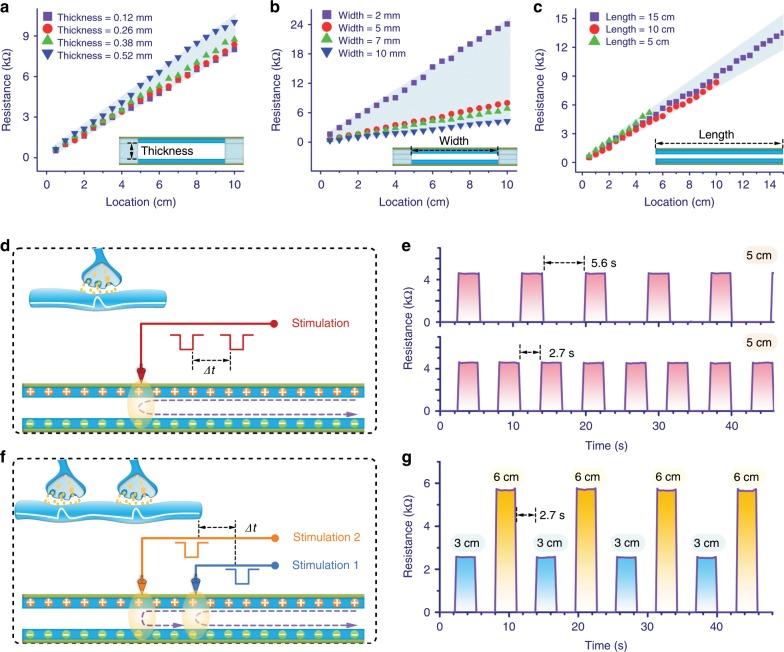
Performance and characteristic of the APT nerve. **a** Influence of the thickness of spacer on the response resistance of the APT nerve with the active width of 5mm. Relationship between the location of mechanical stimulation and the response resistance of the APT nerve with different active **b** width and **c** length when the spacer was 0.12 mm. **d** Diagram of one type of mechanical stimulation applying on the APT nerve. Top left: Schematic of a single synapse. *Δt* is the interval time between adjacent mechanical stimulations. **e** Change in the response resistance of the APT nerve triggered by the mechanical stimulation at different intervals (5.6 s and 2.7 s). The mechanical stimulation was applied at the location of 5 cm of the APT nerve. **f** Diagram of mechanical stimulations applying at different locations (3 cm and 6 cm) of the APT nerve. Top left: Schematic of spatiotemporally dynamical stimulations of two synapses. **g** Spatiotemporally dynamical response of the APT nerve to the mechanical stimulations. The interval was 2.7 s.

To characterize the performance, the influence of the thickness of the spacer on the APT was evaluated (Fig. 2a). The APT nerve was applied by the mechanical stimulation with a blade every 5 mm from the beginning of the electrodes. The result showed that the relationship between the response resistance and the location of the mechanical stimulation was nearly linear. This phenomenon in which the response resistance slightly increased with the increase of the thickness of the spacer was due to incomplete electric contact between the upper and bottom of the active layers (Supplementary Fig. 2). Furthermore, the performance of the APT nerve with different parameters of the active layer was tested (Fig. 2b). Notably, no matter the width of the active layers was changed, the linear relationship between the response resistance and the location of the mechanical stimulation was still maintained. When the mechanical stimulation was applied on the same location of the APT nerve with different width of the active layer, the change in the response resistance was mainly caused by the fact that the resistance of the active layer was modulated by the width. On the other hand, the response resistance of the APT nerve was substantially fixed to the location of the mechanical stimulation when the width of the active layer and the thickness of the spacer were fixed (Fig. 2c). To normalize the size of APT nerve, the thickness of the spacer and the width of the active layer were fixed as 0.12 mm and 5 mm, respectively, unless otherwise noted. The extension of the active length brought a more changeable range in the response resistance of the APT nerve for a broader range of detection and recognition of the mechanical stimulation, which meant more possibilities and diversity for interactive applications based on artificial intelligence.

Most devices would lose their functionality completely after some local damage. Due to the unique architecture of the APT nerve, even if a part of the device was sheared off, the rest was still able to sense mechanical stimulation, transmit signals, and recognize the location of the mechanical stimulation (Supplementary Fig. 3). This was because the response resistance of the APT nerve was only affected by the active length between the location of the mechanical stimulation and the electrodes. Loss of some terminal portion of the APT nerve did not change the connection of the electrodes and the remaining conductive graphite films. This characteristic was similar to the one of the biological sensory nerve that the partial loss of the end did not make a complete loss of neural perception^33^, and was beneficial to reduce some subsequent maintenance or frequent replacement of the device. In addition, since the final step of the device fabrication was to pack two paper-based components face-to-face together by a thin transparent tape, it could effectively isolate the paper substrate from contacting with the outside water. Thus, the as-fabricated APT nerve featured waterproofness and possessed the reproducibility of the response after storing in a humid place (Supplementary Fig. 4).

An electrical or chemical signal propagating along the axon from a neuron to another one needs to pass a biological synapse (Fig. 2d). Stimulation below the detection threshold does not cause the generation of nerve signal for cortical sensation^32^. Analogously, as the sensing mechanism was based on electric contact, there would also be a detection threshold for the APT nerve. Thus, the response threshold that the pressure caused the APT nerve to generate a stable response signal was tested (Supplementary Fig. 5). The result showed that the APT nerve could stably respond to the mechanical stimulation requiring it larger than ~5 kPa. Subsequently, the response of the APT nerve was mainly influenced by the location of the mechanical stimulation. The change in the response of the APT nerve over time to mechanical stimulation was referred to the plasticity of the biological synapse, which was an essential basis for learning. A short-term and interval mechanical stimulation was adopted to study the responses of the APT nerve (Fig. 2e). In order to eliminate a potential pressure deviation from the loading equipment, a loading pressure of large than 5 kPa (as an example, about 10 kPa) was applied on the APT nerve for the test. It could be found that the APT nerve responded fast (<21 ms) to the mechanical stimulation and the mechanosensitive signal disappeared quickly (<21 ms) when the mechanical stimulation stopped (Supplementary Fig. 6). The response of regular fluctuations was similar to the excitation and inhibition of a biological synapse, which was also activated by the external stimulation and then returned to the original state after removing the external stimulation^34,35^. The results from multiple testing with the different intervals (2.7 s and 5.6 s) of the mechanical stimulations indicated the APT nerve was highly robust.

Biologically, the spatiotemporally dynamic logic of neural network is formed by dynamically transmitting postsynaptic potentials from multiple presynapses at intervals (Fig. 2f). Since the response resistance was highly regional differentiated to the location of mechanical stimulation, the APT nerve could also be served as a geometrically hierarchical sensing device to mimic the spatiotemporally dynamic logic of the neural network. For the demonstration, the short-term mechanical stimulations were applied to different locations (3 cm and 6 cm) of the APT nerve at the interval of 2.7 s. Fast and stable changes in the resistance with different amplitudes were generated to respond to the spatiotemporally dynamic mechanical stimulation (Fig. 2g). The response of the APT nerve was analogous to that of neural potentials trigged from different presynapses when spatiotemporally dynamic stimulations were applied on different mechanoreceptors. Thus, a basic spatiotemporally dynamic logic was demonstrated by using the APT nerve. Note that this feature makes the APT nerve potentially useful for time-enhanced ciphers, of which the code changes over time to prevent the case that a hacker gets the correct sequence of code to access to confidential information (Supplementary Fig. 7). Another important consideration for the use of the APT nerve was the stability of the response to mechanical stimulation. The response resistance of the APT nerve under the mechanical stimulation at different locations for a durability test of >10,000 cycles was shown in Supplementary Fig. 8. For realistic applications, follow-up protections would be provided to make the functional nerve device durably work, while it would extend the preparation process, require more materials, increase the costs, and may limit the portability of the functional nerve device. The result showed that the APT nerve without the cumbersome protections possessed high repeatability and durability. Furthermore, it indicated that the as-prepared APT nerve based on the separate electrical double-layers structure provided the prototype sample that would be a beneficial alternative to remove the imperfections of follow-up protections for long-term reliable mechanosensitive interactions towards realistic applications.

### Multifunctional touch interaction of the APT nerve

**Fig. 3:**
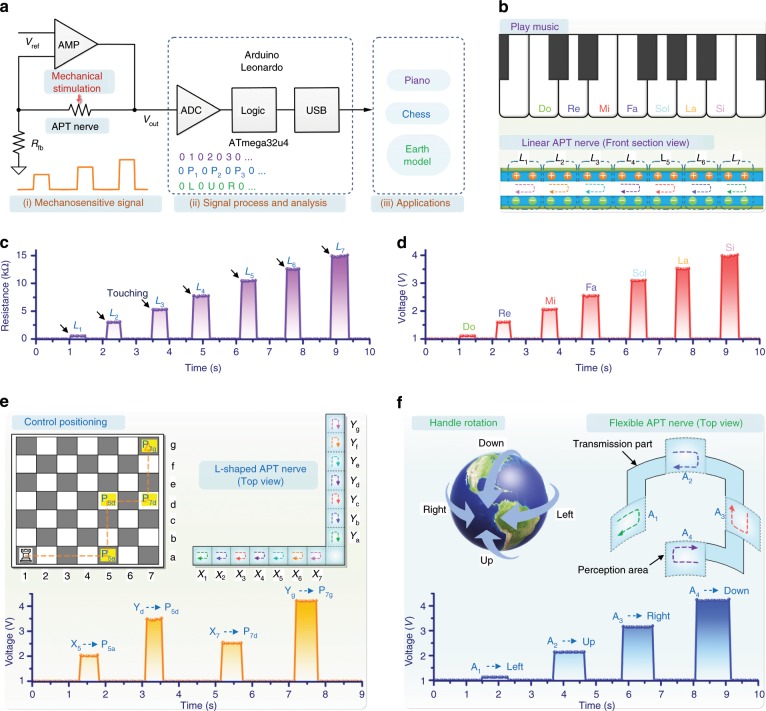
Multifunctional touch interaction of the APT nerve. **a** Circuit schematic to drive the APT nerve for applications. **b** A linear APT nerve used for playing music. The tonic solfa of Do, Re, Mi, Fa, Sol, La, and Si would be produced by touching corresponding segments of the APT nerve. **c** Response resistance of the linear APT nerve when touching different segments. **d** Change in the voltage producing corresponding tonic solfa. **e** An L-shaped APT nerve used for controlling the position of a chess piece in a two-dimensional plane. The horizontal branch controlled the horizontal movement of the chess piece and the other branch controlled the movement of the chess piece on the vertical axis. Bottom: Change in the voltage when touching different segments. **f** Demonstration of handling the rotation of an earth model by using a flexible APT nerve with a square shape. Each perception area was 10 × 10 mm^2^. Bottom: Touching different perception areas resulting in the changes in the voltage.

Next, as a proof-of-concept, the APT nerve was used to implement multifunctional touch interaction (Fig. 3). A driver circuit was designed to communicate the APT nerve with a computer. Figure 3a shows the circuit schematic to drive the APT nerve. The first stage was a linear transform from resistance into voltage so that it could be further converted into the digital domain by an analog to digital converter (ADC). This convertor was composed of an operational amplifier with a feedback resistor network. The output voltage (*V*_*out*_) was proportional to the response resistance (*R*_*total*_), which was shown in the following formula:

2 $$V_{out} = \left( {1 + R_{total}/R_{fb}} \right) \times V_{ref}$$

where *R*_*fb*_ and *V*_*ref*_ were the feedback resistor and the reference voltage, respectively. The analog output of the driver circuit was digitalized and synchronized by a microcontroller development board (Arduino Leonardo), which integrated a low-power microcontroller chip (ATmega32u4) as its central processor. The microcontroller development board had total number of 12 internal on-chip analog-to-digital convertors (ADC), and one of them was connected to the analog output voltage as its input sensing signal. Hence, the analog voltage was digitalized into the range of 0–1023 by such 10-bit ADC in a sample frequency of 100 Hz. There was a look up table built inside the microcontroller so as to map the digital value to the location of the mechanical stimulation. Furthermore, there was a built-in universal-serial-bus (USB) interface on this development board, which enabled microcontroller to communicate with personal computers (PC). Based on the digital value of the output voltage, this development board could send corresponding pre-defined commands to the PC side. Since there would be customized applications developed on the computer side, actions would be performed as the responses based on the received command. It should be noted that during the test of finger touch, the contact area between the fingertip and the APT nerve may change somewhat, resulting in a slight change in the contact area of the upper and bottom active layers. However, the influence of this change on the response resistance was limited. Each response resistance was still stable even with some negligible fluctuations. It could be directly converted into voltage, which was recognized by the microcontroller. Thus, this process did not need additional denoising. On the other hand, when the finger touch was removed from the APT nerve, the response resistance quickly disappeared. Accordingly, the output voltage would return to the original state. Therefore, baseline tracking processing was not required to prevent baseline drift.

Figure 3b described a linear APT nerve with the active length of 140 mm that was virtually divided into seven segments, namely L_1_, L_2_, L_3_, L_4_, L_5_, L_6_, and L_7_, corresponding to the tonic solfa of Do, Re, Mi, Fa, Sol, La, and Si. Each perception segment was 5 × 20 mm^2^. When a finger was touching a certain segment, the response resistance of the linear APT nerve was quickly generated (Fig. 3c) and maintained. Accordingly, the output voltage of the driver circuit was changed (Fig. 3d). It could be found that these changes in the output voltage were different from each other so that the microcontroller could easily identify them and made the virtual piano produce the corresponding sounds. Vivid playing music by a finger touching on the linear APT nerve was shown in Supplementary Movie 1.

Due to the scalability of the preparation process and the universality of the design principle, in addition to the linear strip shape, the architecture of the APT nerve could be designed to be L-shaped. The second demonstration was that the L-shaped APT nerve was designed and fabricated to control positioning in a two-dimensional plane (Fig. 3e and Supplementary Movie 2). The detailed parameter of the L-shaped APT nerve was shown in Supplementary Fig. 9a. The horizontal branch of the L-shaped APT nerve was virtually divided into seven segments, namely X_1_, X_2_, X_3_, X_4_, X_5_, X_6_, and X_7_, and would control the horizontal movement of a chess piece, which was created and could move in a 7 × 7 grid on PC. Similarly, the other seven segments from the vertical branch, naming as Y_1_, Y_2_, Y_3_, Y_4_, Y_5_, Y_6_, and Y_7_, were used to control the movement of the chess piece on the vertical axis. Based on this, the movement of the chess piece in any position in the two-dimensional plane would be realized by touching the corresponding segment of the L-shaped APT nerve. For example, when a finger touched X_5_, Y_d_, X_7_, and Y_g_ in sequence, the response resistance of the L-shaped APT nerve would change the output voltage of the driver circuit (bottom of Fig. 3e). According to different voltages, the microcontroller would send the machine-recognized codes that moved the chess piece from P_1a_ to P_7g_ through P_5a_, P_5d_, and P_7d_. It should be noted that the L-shaped APT nerve only needed one channel so that contributed to simplify the signal processing and avoid the potential problems of the integration of numerous sensing elements, such as intricate interconnections, complicated structure, and interference in signal transmission. Alternatively, an S-shaped APT nerve could be also designed and would be effective for the positioning control in the two-dimensional plane (Supplementary Fig. 9b).

Furthermore, in order to demonstrate the proposed APT nerve still worked well under bending state, we designed and fabricated a flexible APT nerve with a square shape and built a free-rotating earth model (Fig. 3f). Each perception area of the flexible APT nerve was 10 × 10 mm^2^ (Supplementary Fig. 9c) and named as A_1_, A_2_, A_3_, and A_4_. Touching different perception areas would cause the change in the output voltage of the driver circuit (bottom of Fig. 3f). Thereupon, the earth model could be rotated up, down, left, and right by touching the corresponding perception area of A_2_, A_4_, A_1_, and A_3_. The response resistance of the flexible APT nerve was generally stable by finger touch no matter it was in the flat state or the bending state (Supplementary Fig. 10). Supplementary Movie 3 shows that we fit the flexible APT nerve on a bottle with a radius of ~2.7 cm to handle the rotation of the earth model. This result demonstrated the proposed APT nerve featured flexibility that was similar to the biological nerve.

Although the above three demonstrations were limited to one finger touch, the results indicated the method provided here and the proposed sensing mechanism could make the APT nerve available to be designed and fabricated into a variety of architectures as needed, which provided essential prototypes for building future all-in-one APT neural networks. In addition, considering the nonlinear relationship between the biological sensory neuron and the external mechanical stimulation, the linear relationship between the input mechanical stimulation and the output signal from the APT nerve could be converted to be nonlinear as needed by transduction circuits or taking use of microcontroller in a software manner (Supplementary Note 1).

### The APT nerve for perceptual learning

**Fig. 4:**
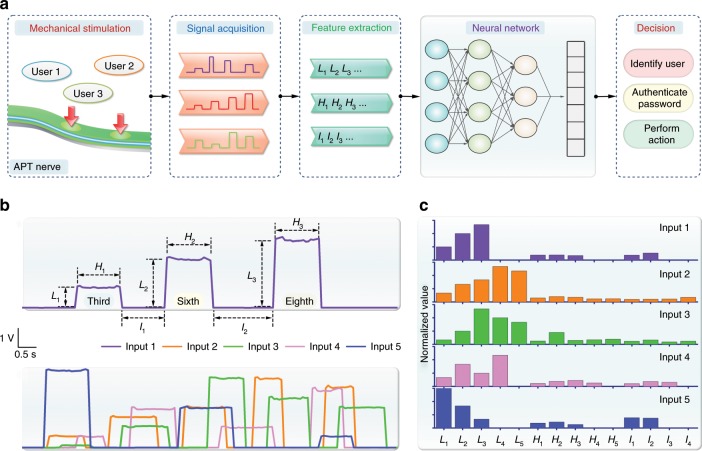
The APT nerve for perceptual learning. **a** Schematic diagram of the machine learning based on the APT nerve, including recognition of mechanical stimulation, signal acquisition, feature extraction, decision of neural network. **b** Mechanosensitive signals from the APT nerve corresponding to different input information. The touching characteristic parameters included the touching location (*L*), holding time (*H*), latency interval (*I*) to characterize different mechanical stimulation within a set time (10 s). **c** Three-factor feature map based on the touching characteristic parameters extracted from **b**.

The human brain learns and responds to different stimulation by processing and analyzing the signals from the biological sensory nerves. Like the functions of biological sensory nerves, the proposed APT nerve had the potential to target human-machine interaction based on artificial intelligence, such as user identification, feedback control of prosthesis, and intelligent action of a robot. Figure 4a illustrates a basic process flow combined with algorithmic analysis of artificial intelligence. The mechanical stimulations from different users' touching were firstly converted to digital signals by the APT nerve and driver circuit. Through a logical operation, the features of the mechanosensitive signals would be extracted and then put into an artificial neural network to decide to respond or not.

As a simple demonstration of the functionality, the APT nerve with the active length of 180 mm was virtually and evenly divided into nine perception segments. In this way, different perception segments stimulated by a finger could be distinguished by the magnitude of response signals. Above studies have shown that the APT nerve possesses a spatiotemporal resolution function like that of biological nerve. Thus, we could set three touching characteristic parameters, including touching location (*L*), holding time (*H*), latency interval (*I*), to characterize different mechanical stimulation of finger touch within a set time (10 s). For example, when we subsequently touched third, sixth, and eighth perception segments, the mechanosensitive signal from the APT nerve was generated quickly and showed the corresponding magnitude (Fig. 4b upper). Different mechanosensitive signals would also be generated to reflect the personal input information from other users through the APT nerve (Fig. 4b lower). Subsequently, the touching characteristic parameters were extracted from the mechanosensitive signals by using logical analysis. The results indicated that these different inputs could be characterized by the three-factor feature map (Fig. 4c). Therefore, through many times of sampling and analysis, we could get a touching feature database. After that, the *k*-nearest neighbors algorithm^36^ was used for the classification. The main idea of this algorithm was that if the majority touching characteristic parameters from one unknown input were most similar to the ones from the several known inputs, the unknown input and the several known inputs belonged to the same category. In details, after that multiple inputs from the authorized users were detected, a database of the input characteristics from the authorized users would be built. Thereafter, when a new input from an unknown user was detected, the characteristic parameters of this input would be compared with the database. If the similarity of input characteristics between them researched 90% (system-defined) or more, the system could judge that the unknown input was from an authorized user, otherwise, it was from an unauthorized user. The more data got, the result would be more accurate. The backend procedures would use this result to make appropriate feedback for the interaction.

## Discussion

In this work, we have introduced an all-in-one bionic sensory nerve that is called as APT nerve and demonstrate its integrated functions of detecting mechanical stimulation, consuming no energy when no mechanical stimulation happens, recognizing the location of mechanical stimulation, and transmitting mechanosensitive signal for mimicking the main functions of the biological sensory neural system. Although the artificial sensory neural system is still progressing in the infancy stage, by discovering the performance and characteristic of the proposed APT nerve, we have exploited the remarkable multifunctional touch interactions of the APT nerve, including playing music, controlling positioning in the two-dimensional plane, and free handling rotation. The as-desired APT nerve was based on the separate electrical double-layers structure that addressed the critical challenges in intricate interconnections, complicated structure, and interference in signal transmission. The stable and high performance of the APT nerve ensured that the analysis and processing of data could performed without additional denoising and baseline tracking that would greatly simplify logic circuit design and fabrication processes. Due to the non-pixelated sensing characteristic, the APT nerve could be virtually divided into multiple perception segments according to the need of interactive applications towards socially intelligent robotics. Furthermore, the feature of spatiotemporally dynamic logic made the APT nerve potential for perceptual learning, which would genuinely promote neuroprosthetics and robotics into sophistication. Ultra-robustness and excellent durability of >10,000 cyclical tests enabled the APT nerve to operate for highly stable and reliable mechanosensitive interactions and to avoid frequent maintenance. The separate electrical double-layers structure made sure that the APT nerve featured no energy consumption when there was no mechanical stimulation, which could achieve long-term work. The APT nerve could operate perfectly in the flat and bending states. High flexibility and unique cuttability allowed integrating the APT nerve with the human body to be comfortable as needed. Geometrically hierarchical sensing, rapid response (<21 ms) and high variability in shape endowed the great potential of the APT nerve for multifunctional touch interactions. We believe this APT nerve provided an aspirational and significant alternative to construct artificial sensory neural system for advancing socially intelligent robotics and smart prosthetics.

The method provided here made the APT nerve available for large-scale fabrication and allows the APT nerve to be designed into a wide variety of prototype devices. The achievement of the flexibility of the APT nerve attributed to the fiber-based paper substrate. Because of the water-absorbent characteristic of the substrate, a thin transparent tape used to fabricate the device would make the APT nerve waterproof to interact with sweaty fingers. The as-prepared APT nerve could operate perfectly in both flat and bending states due to the opposite changes in the resistance of the two active layers when being bent. In order to realize the stretchability of the APT nerve for a wider range of future applications, organic materials, such as poly(3,4-ethylenedioxythiophene):poly(styrenesulfonate)^37^, nitrile butadiene rubber coating with carbon nanotubes and silver^38^, and poly(styrene-block-butadiene-block-styrene) absorbed with silver nanoparticles^39^, may be a feasible alternative due to their stretchability and conductivity, while it needs further exploration and research to achieve the stable signal output of the APT nerve during stretching. Analogous to the function of the interneuron in the spinal cord, the APT nerve was limited to recognize one mechanical stimulation per time effectively based on the separate electrical double-layers structure. Nevertheless, it is thought-provoking to further consider and provide a feasible strategy to make the device’s structure still extremely simple and highly effective while the APT nerve can sense multiple mechanical stimulations and recognize their locations. Besides, there is some potentially effective noise, which is random or unpredictable fluctuations and disturbances, in the biological neural system, even without any mechanical stimulation^40^. And yet for all that, the random disturbances of signals in the proposed artificial sensory neural system may cause the microcontroller to misjudge and issue wrong commands. Currently, we adopted the separate electrical double-layers structure to reduce noise so that the system could clearly perform the actions as we wanted. Continuous exploration would be conducted to make the bionic devices towards realistic applications. In the future, the combination of different functional materials for fabricating devices will enable the APT nerve to possess versatile sensations, such as temperature, humidity, and even light, towards human-machine hybrid enhanced intelligence.

## Methods

### Fabrication of the APT nerve

Firstly, two pieces of adhesive tapes (Scotch Magic^TM^ Tape 810#, 3 M Inc.) were pasted on a piece of graph paper (No.704, QIANCAILE) to form a specified hollow pattern. Typically, the hollow area was 5 × 140 mm^2^. An 8B pencil (No. 6841, Deli Company) was then adopted to draw on the hollow area of the paper substrate. Several times of drawing was performed to make the hollow area fully filled with graphite. After peeling off one layer of the tape-based pattern, a well-regulated and as-designed graphite-based film was formed and served as the conductive path. Subsequently, the new adhesive tapes were attached on both sides of the graphite strip along the long axis, which were served as the spacer. The typical thickness of the spacer was 0.12 mm. The conductive line was led out at one end of the long axis of the conductive path by silver paint (SPI-PAINT, Structure Probe, Inc.). The above steps fabricated a basic part of the APT nerve. Another part of the APT nerve was also prepared in the same steps, while its pattern was mirror-symmetrical to the one of the previous part. Finally, the as-prepared parts were face-to-face put together by the adhesive tape and assembled into the APT nerve. The adhesive tape used here also would provide the waterproofness of the APT nerve.

### Signal processing circuit

During the test of finger touch, a response resistance would be generated by touching a part of the APT nerve, of which the active layer was 5 × 140 mm^2^ and the thickness of the spacer was 0.12 mm. After initial evaluation, the minimum response resistance was 0.5 kΩ that was generated by touching the portion that was closest to the electrodes. Oppositely, when the finger touched the portion that was farthest from the electrodes, the maximum response resistance was generated and its value was 15 kΩ. Thus, the values of other response resistances, which were generated by touching different portions of the APT nerve, would be within from 0.5 to 15 kΩ, but each of them was stable and not with an observable fluctuation. In order to make the output voltage within 5 V, the feedback resistor *R*_*fb*_ was selected with 5 kΩ and the reference voltage *V*_*ref*_ was configured as 1 V. The output voltage *V*_*out*_ swung in this circuit was 2.9 V, which linearly mapped the response resistance of the proposed APT nerve. The amplifier (AMP) model we used was LM741 designed by Texas Instruments. In particular, regarding to the L-shaped APT nerve, as the active length became longer, the maximum response resistance increased up to 33 kΩ. So, the feedback resistor was selected with 10 kΩ.

### Characterization and measurement

Scanning electron microscopy (JEOL, JSM 6360) was employed to observe the surface morphology of the graph paper and the graphite-based conductive film. The digital multimeters (UNIT UT39B and Agilent 34461A) were used to detect and record the response resistance of the APT nerve. The customized actuator (Beijing Times Brilliant Electric Technology Co., Ltd.) applied the dynamical pressure on the APT nerve for the tests. The applied force was calibrated by the standard force sensor (Bengbu Sensors System Engineering Co., Ltd., JHBM-7). The thickness of the spacer was measured by the vernier caliper (HANS.w, HS1044A).

## Supplementary information

Supplementary Information

Description of Additional Supplementary Files

Supplementary Movie 1

Supplementary Movie 2

Supplementary Movie 3

## Data Availability

The data that support the findings of this study are available from the corresponding author upon reasonable request.
