# Supplementary material for: A bioinspired analogous nerve towards artificial intelligence
Source: Nat Commun. 2020 Jan 14;11:268. doi: 10.1038/s41467-019-14214-x (PMC6959309; doi:10.1038/s41467-019-14214-x)
Supplement: Supplementary file 1 — Supplementary Information [file 41467_2019_14214_MOESM1_ESM.pdf]

Supplementary Information for

A Bioinspired Analogous Nerve towards Artificial Intelligence

Liao et al.

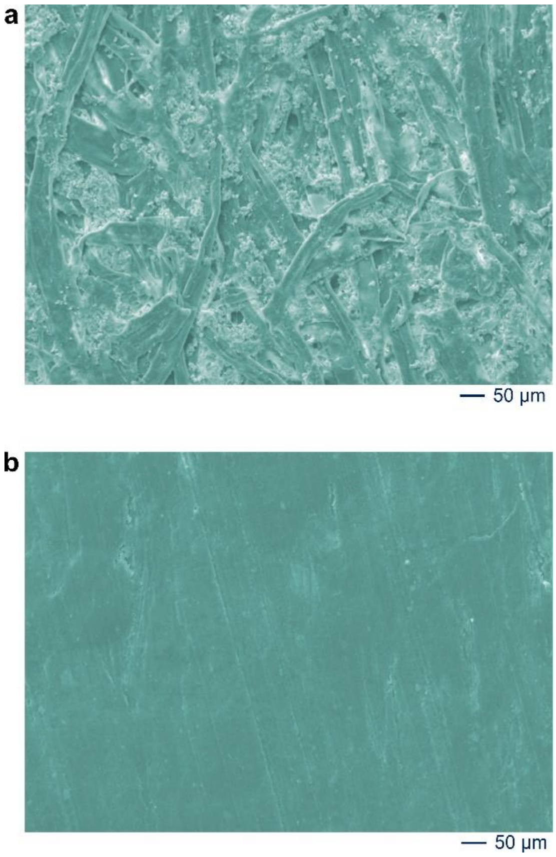

Supplementary Figure 1. Scanning electron microscopy images of graph paper and graphite film. (**a**) Scanning electron microscopy image of graph paper, which shows the porous structure that was a benefit to depositing graphite. It could be found that the graph paper was composited of substantial one-dimensional cellulose fibers, which stacked together to form the porous structure. (**b**) Scanning electron microscopy image of graphite film. The graphite film was deposited on a piece of graph paper and featured smoothness. The smooth surface of the graphite film was conducive to making the upper and bottom active layers contact close only by applying light pressure. When the pressure was removed, the upper and bottom active layers could be quickly separated to avoid residual contact.

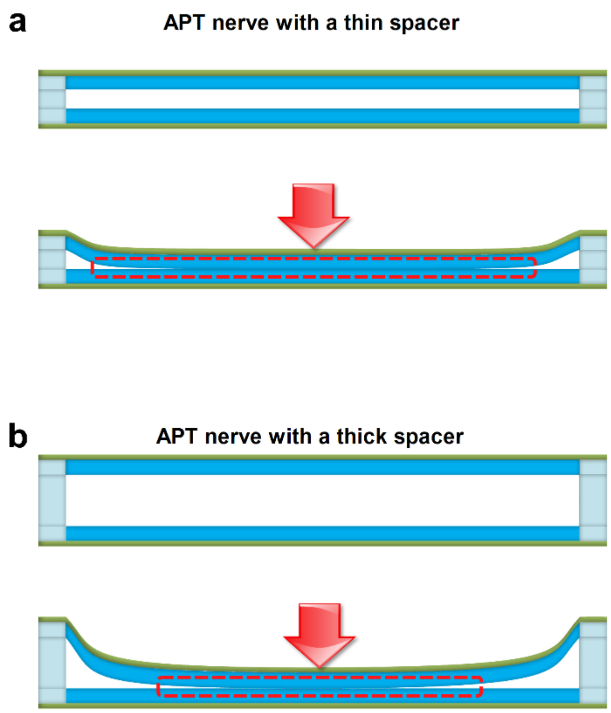

Supplementary Figure 2. Schematic of incomplete electric contact. (**a**) The morphologies of the APT nerve with a thin spacer before and after applying mechanical stimulation. (**b**) The morphologies of the APT nerve with a thin spacer before and after applying mechanical stimulation. All the morphologies of the APT nerve were in the right section view. The contact area of the upper and bottom active layers was pointed out by a red dotted frame. Less contact between the upper and bottom active layers happened under pressure when the APT nerve consisted of a thick spacer. This less contact would cause a slight increase in the response resistance, which was because it was difficult for the movement of electrons in a small contact area.

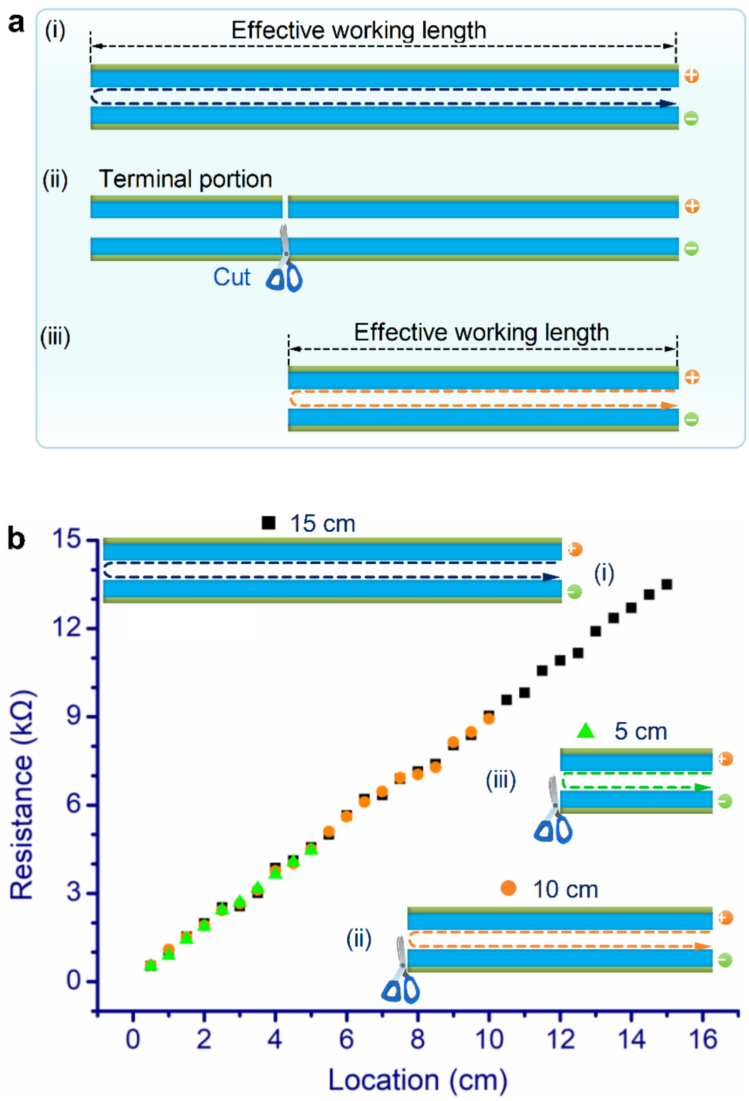

Supplementary Figure 3. Cuttability of the APT nerve. (**a**) Schematic of the cuttability. Since the APT nerve was mainly made of the cuttable materials, the as-fabricated device could be easily cut by scissors. In our design, the electrodes were at the same side of the APT nerve. Therefore, after cutting, although the active length of the APT nerve was reduced, the rest portion of the APT nerve with the electrodes could still work effectively. (**b**) The relationship between the response resistance and the location of the mechanical stimulation before and after a part of the APT nerve was sheared off. The result showed that the linear relationship and the magnitude of the response resistance of the cut APT nerve were the same as that of the original one, even when the APT nerve was cut several times. The cut APT nerve was still able to sense mechanical stimulation, transmit signals, and recognize the location of the mechanical stimulation.

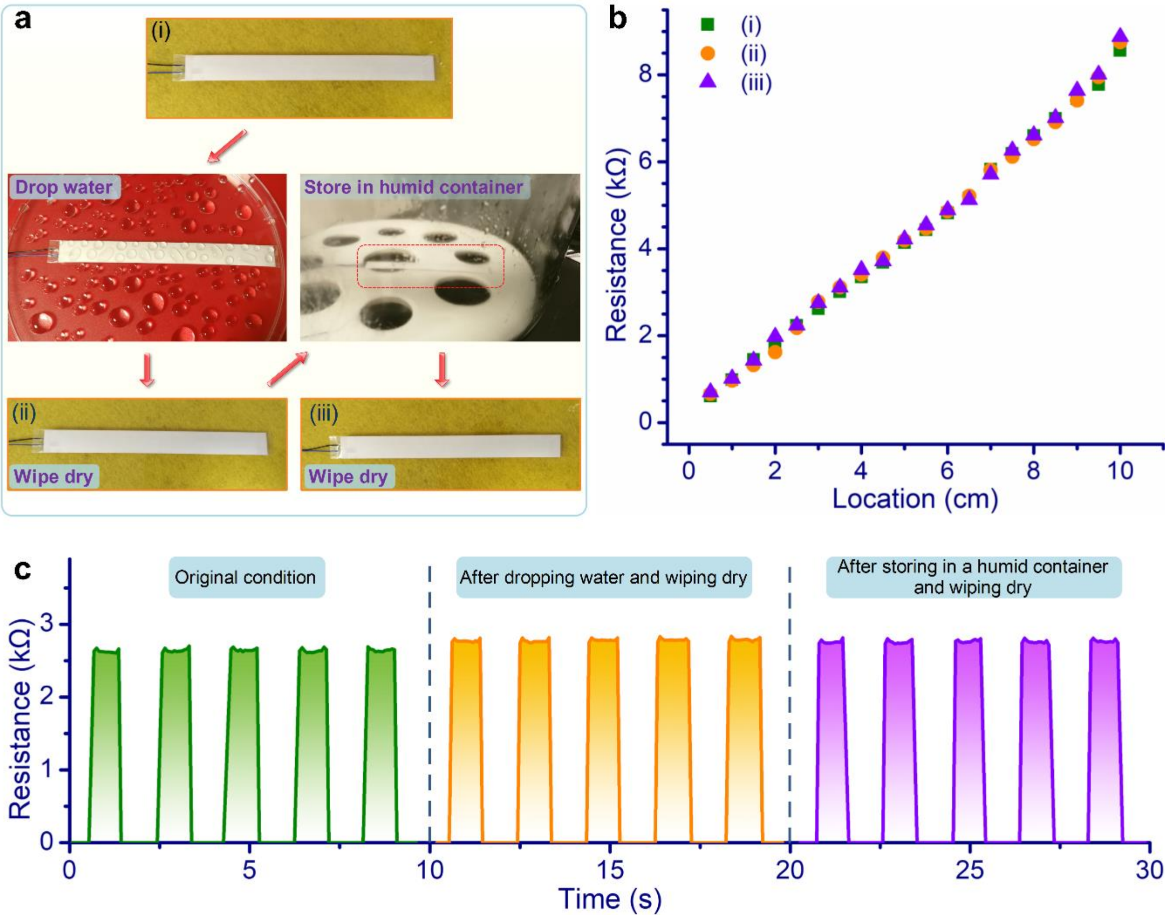

Supplementary Figure 4. Waterproofness of the APT nerve. (**a**) Photographs of the APT nerve (i) in the original condition, (ii) after dropping water and wiping dry, and (iii) after storing in a humid container and wiping dry. The state of the APT nerve covered by the water droplets and the presence in the wet container lasted for 5 minutes and 12 hours, respectively. The APT nerve stored in the humid container was pointed out by a red dotted box. Tissue paper was used to wipe the APT nerve dry. (**b**) Response resistance of the APT nerve in different conditions. As the thin transparent tape was effective to isolate the paper substrate of the APT nerve from contacting with the outside water, the response resistances of the APT nerve in different state were almost same. (c) Multiple cyclical tests of the APT nerve in different conditions. The mechanical stimulation was applied at the location of 3 cm of the APT nerve. The reproducibility of the responses confirmed that the APT nerve could continue to operate even after dropping water and storing in a humid container.

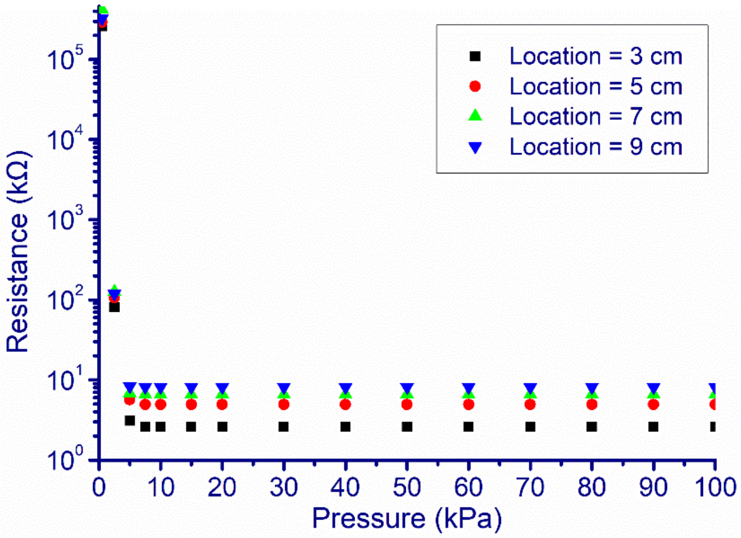

Supplementary Figure 5. Relationship of the pressure and response resistance. As the sensing mechanism of the APT nerve was based on electric contact, the response resistance would be influenced by the contact area between the upper and bottom active layers, which was changed by the pressure. The greater the pressure applied, the larger the contact area, and the smaller the response resistance. Since the surfaces of the upper and bottom active layers were smooth, a pressure of 5 kPa made the contact saturated. Thus, the response resistance would be fixed to the pressure of > 5 kPa and the APT nerve could stably respond to the mechanical stimulation. The different magnitude of the fixed response resistance would be used to recognize the location of the mechanical stimulation.

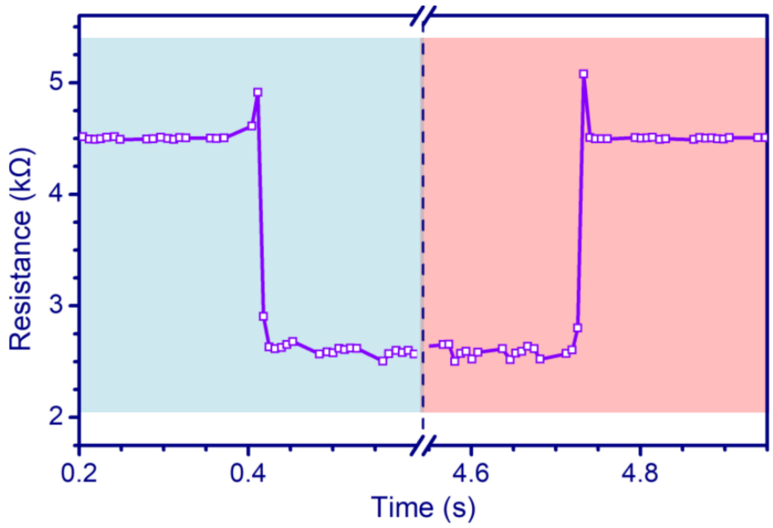

Supplementary Figure 6. Response time of the APT nerve. For the test, the APT nerve was connected in parallel with a resistor of 4.5 kΩ. The mechanical stimulation was applied at the location of ~ 5 cm of the APT nerve. It could be found that the APT nerve responded fast (< 21 ms) to the mechanical stimulation and the mechanosensitive signal disappeared quickly (< 21 ms) when the mechanical stimulation stopped.

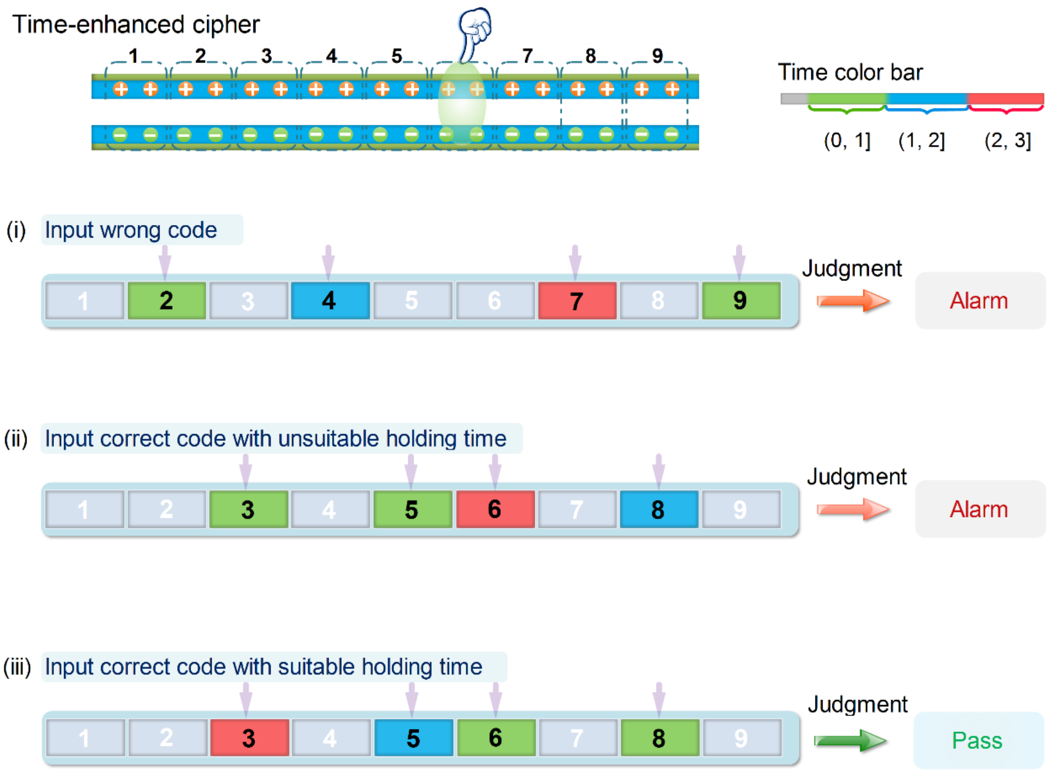

Supplementary Figure 7. Schematic of time-enhanced ciphers based on the APT nerve. The APT nerve was virtually divided into nine perception segments. Each perception segments corresponded to one number. Since the APT nerve responded stably to the mechanical stimulation, the holding time of the mechanical stimulation could be set and reflected by different colors, such as gray (no touch), green (less than 1 s holding), blue (holding between 1 s - 2 s), red (holding between 2 s - 3 s). Therefore, the correctness of the code needed to consider not only the sequence of the mechanical stimulation but also the holding time of the mechanical stimulation. For example, inputting wrong code would make the alarm (i), which was the same function as an ordinary cipher. However, when the correct sequence of the code, which was 3 (green), 5 (green), 6 (red), 8 (blue), was input into the APT nerve, the alarm would be still triggered due to the unsuitable holding time of the mechanical stimulation (ii). Only when the correct sequence of the code was input with the suitable holding time of the mechanical stimulation, set as 3 (red), 5 (blue), 6 (green), 8 (green), into the APT nerve, the alarm would not be triggered (iii). So, even if a hacker got the correct sequence of code, the time-enhanced cipher would prevent the leakage of confidential information.

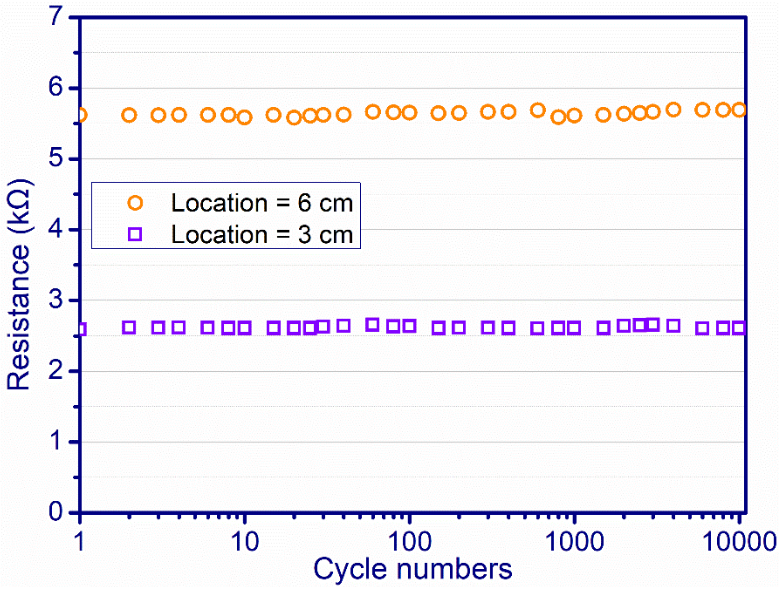

Supplementary Figure 8. Durability tests of the APT nerve. The response resistance of the APT nerve under the mechanical stimulation at different locations (3 cm and 6 cm) for a durability test of >10,000 cycles. The response resistance was recorded by a digital multimeter at intervals. It could be found that the response resistance was almost stable during the test of the same mechanical stimulation.

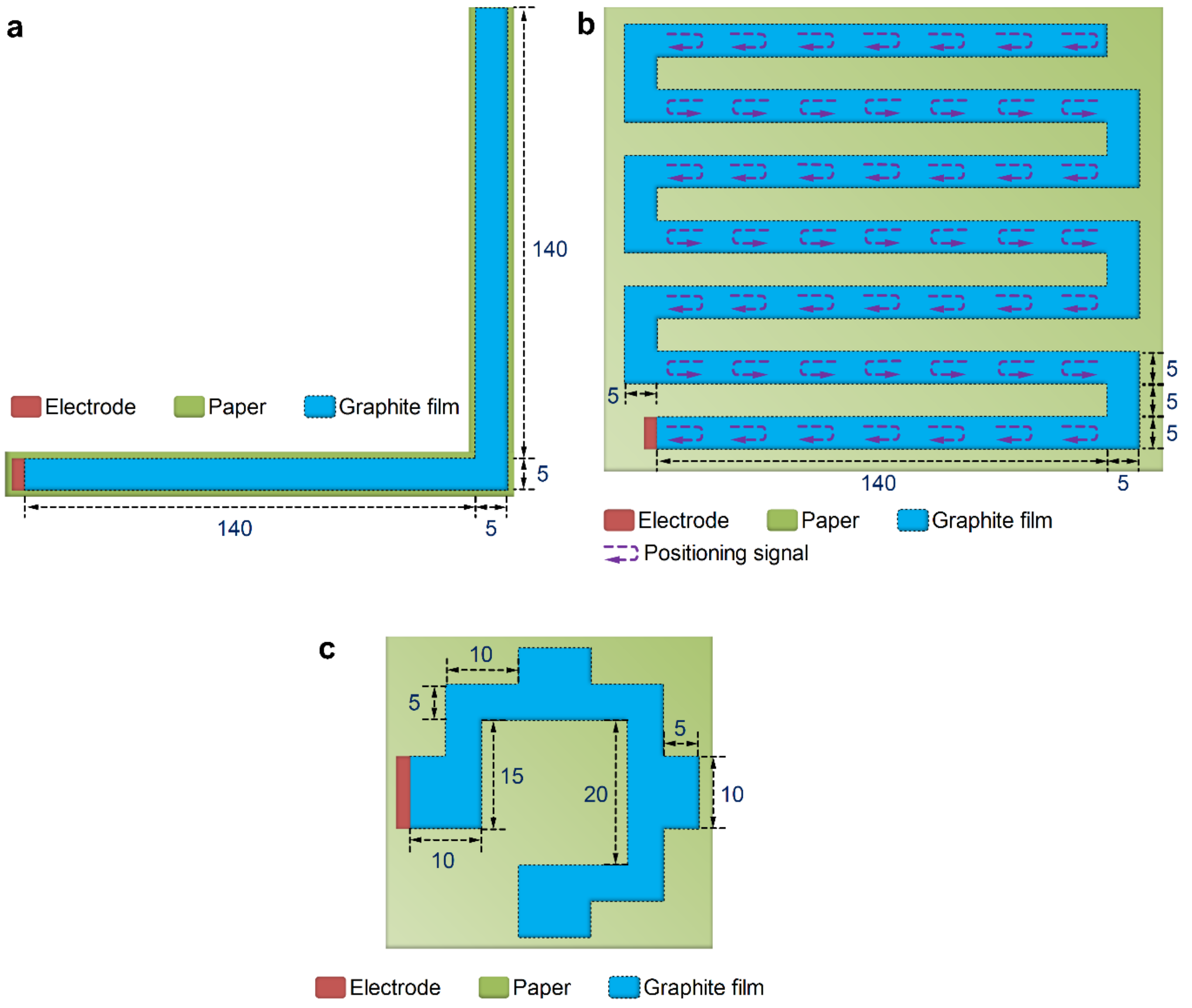

Supplementary Figure 9. Structural parameters of the (a) L-shaped, (b) S-shaped, and (c) square APT nerves. All the morphologies of the APT nerve were in the top view. The unit in the figure is millimeter. The thickness of the spacer was 0.12 mm. Two parts, each of which was the paper-based substrate combined with conductive graphite film, were mirror-symmetrical for the fabrication of these APT nerves. For the L-shaped and S-shaped APT nerves, each virtual perception segment was 5 × 20 mm^2. For the square APT nerve, each perception area was 10 × 10 mm^2.

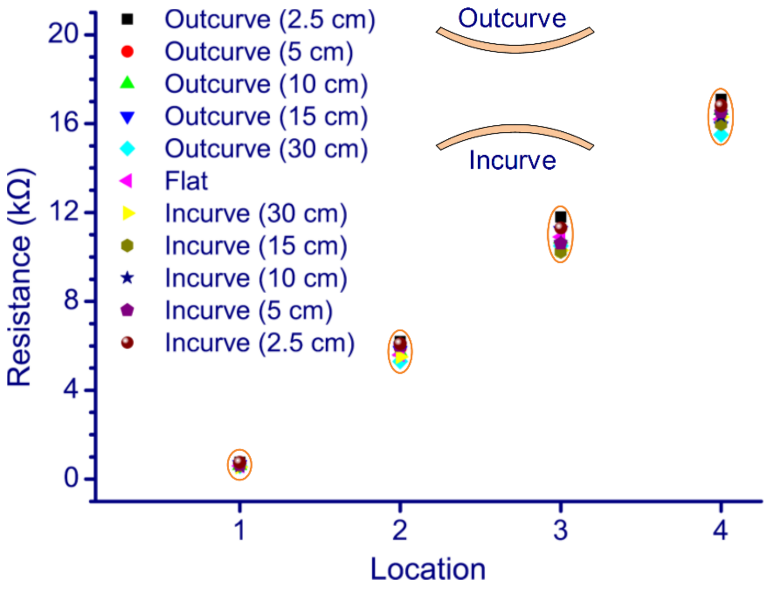

Supplementary Figure 10. Relationship of the response resistance and location. The response resistance of the flexible APT nerve was generally stable by finger touch, whether it was in the flat state or the bending states with different radiuses. Although the resistance of the conductive graphite film was sensitive to the strain, the resistance that could be changed by the bending strain was relatively small since the bending strain was basically less than 1%. On the other hand, when the device was bent, the upper and lower active layers produced opposite strains that were tensile strain and compressive strain, respectively. Thus, the changes in the resistance of the two active layers were exactly opposite. One of the active layers was in the case that the resistance increased, while the resistance of the other active layer decreased. As a result, the overall resistance of the flexible APT nerve was relatively stable when a finger touched the same perception area and thus, the flexible APT nerve could operate in the bending state.

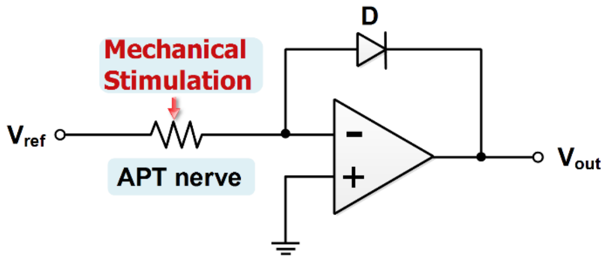

Supplementary Figure 11. Circuit schematic for realizing a non-linear response. V_{ref}, V_{out} and D represent the input voltage, the output voltage and the diode, respectively.

Supplementary Note 1. Nonlinear transformation of linear relations.

In our prototype, the input signal is the transient resistance from the APT nerve, the output signal is represented as an analog voltage. A typical and common example to realize a non-linear response for nature signals is the logarithmic circuit based on an operational amplifier, which is drawn in the circuit schematic of **Supplementary Figure 11**.

In this circuit, if the input voltage (V_{ref}) is fixed in a given value, then the output voltage (V_{out}) has a logarithmic response with the response resistance (R_{total}), which is given as following:

$$V_{out} = -V_t \ln\left[V_{ref}/(I_s R_{total})\right] \quad \text{(S1)}$$

I_s and V_t correspond to the saturation current and thermal voltage of the diode (*D*) respectively, and these values are fixed as constants after fabrication.

In fact, there is another more efficient way to implement such non-linear transformation by taking use of microcontroller in a software manner. For example, in the prototypes reported in this paper, the output voltage has a linear relationship with the response resistance from the APT nerve. Since such analog voltage has been digitalized by the microcontroller with an on-chip ADC, users can further make a non-linear transformation on its digitalized value by making a non-linear computation in the software. Compared to the hardware method, such software approach may be slower as it involves multiple times of multiplication. However, if users can build-up a look up table for such computation, and store the pre-computed output values on the memory in advance, the non-linear transformation can be speed-up significantly by simply indexing the look-up table according to the digital value from the output voltage. This process will save a lot of power and computation resources and can implement from the input signal and eventual output signal at the same time.
