# Supplementary material for: A bioinspired analogous nerve towards artificial intelligence
Source: Nat Commun. 2020 Jan 14;11:268. doi: 10.1038/s41467-019-14214-x (PMC6959309; doi:10.1038/s41467-019-14214-x)
Supplement: Supplementary file 2 — Description of Additional Supplementary Files [file 41467_2019_14214_MOESM2_ESM.pdf]

Description of Additional Supplementary Files

File Name: Supplementary Movie 1
Description: Demonstration of a linear APT nerve for playing music.

File Name: Supplementary Movie 2
Description: Demonstration of a L-shaped APT nerve for controlling positioning.

File Name: Supplementary Movie 3
Description: Demonstration of a flexible APT nerve fit on a bottle for handling rotation of an earth model.
